# The Complexity of Standing Postural Control in Older Adults: A Modified Detrended Fluctuation Analysis Based upon the Empirical Mode Decomposition Algorithm

**DOI:** 10.1371/journal.pone.0062585

**Published:** 2013-05-01

**Authors:** Junhong Zhou, Brad Manor, Dongdong Liu, Kun Hu, Jue Zhang, Jing Fang

**Affiliations:** 1 Academy for Advanced Interdisciplinary Studies, Peking University, Beijing, China; 2 Divisions of Gerontology, Beth Israel Deaconess Medical Center, Harvard Medical School, Boston, Massachusetts, United States of America; 3 College of Engineering, Peking University, Beijing, China; 4 Medical Biodynamics Program, Division of Sleep Medicine, Brigham and Women’s Hospital/Harvard Medical School, Boston, Massachusetts, United States of America; 5 Center for Dynamical Biomarkers and Translational Medicine, National Central University, Chungli, Taiwan; University of Warwick, United Kingdom

## Abstract

Human aging into senescence diminishes the capacity of the postural control system to adapt to the stressors of everyday life. Diminished adaptive capacity may be reflected by a loss of the fractal-like, multiscale complexity within the dynamics of standing postural sway (i.e., center-of-pressure, COP). We therefore studied the relationship between COP complexity and adaptive capacity in 22 older and 22 younger healthy adults. COP magnitude dynamics were assessed from raw data during quiet standing with eyes open and closed, and complexity was quantified with a new technique termed empirical mode decomposition embedded detrended fluctuation analysis (EMD-DFA). Adaptive capacity of the postural control system was assessed with the sharpened Romberg test. As compared to traditional DFA, EMD-DFA more accurately identified trends in COP data with intrinsic scales and produced short and long-term scaling exponents (i.e., α_Short_, α_Long_) with greater reliability. The fractal-like properties of COP fluctuations were time-scale dependent and highly complex (i.e., α_Short_ values were close to one) over relatively short time scales. As compared to younger adults, older adults demonstrated lower short-term COP complexity (i.e., greater α_Short_ values) in both visual conditions (p>0.001). Closing the eyes decreased short-term COP complexity, yet this decrease was greater in older compared to younger adults (p<0.001). In older adults, those with higher short-term COP complexity exhibited better adaptive capacity as quantified by Romberg test performance (r^2^ = 0.38, p<0.001). These results indicate that an age-related loss of COP complexity of magnitude series may reflect a clinically important reduction in postural control system functionality as a new biomarker.

## Introduction

Biological aging is commonly associated with a degradation or breakdown in the complex dynamics of spontaneous physiological fluctuations, such as the fractal patterns (i.e., similar temporal structure at different time scales) in gait and motor activity [1]. Theses dynamic patterns are intrinsic and believed to arise from the network of neuro-physiological control nodes that interact over multiple time scales to regulate behavior and physiology [2], [3], [4]. Thus, an age-related loss of ‘complexity’ may reflect alteration within the neurophysiological control network and a corresponding diminished capacity of the organism to adapt to the innumerable stressors in everyday life [5].

The human postural control system enables bipedal stance along with the capacity to adapt to more stressful conditions such as standing on one leg, completing a cognitive task or reaching for an object [6]. This control system comprises a host of sensory elements integrated with spinal, supraspinal and peripheral motor circuitry [7]. When standing quietly, the dynamics of postural sway–as most commonly estimated by center-of-pressure (COP) fluctuations beneath the feet–are complex [8]. For instance, COP fluctuations possess robust fractal patterns at time scales from milliseconds to minutes [9]. Within the older adult population, sensory impairments [10], [11], frailty [12] and a history of falling have each been linked to diminished COP complexity. However, the effects of normal biological aging (i.e., without abnormal sensory impairments, frailty, or history of falls) on COP complexity are less clear, and the relationship between COP complexity and the capacity to adapt to stressors has not been established.

Numerous metrics have been proposed to quantify the complex characteristics of postural sway dynamics [2], [13], [14], [15]. One metric is based on the assessment of fractal correlations in the *magnitude* of COP fluctuations. In other words, instead of examining the original anterioposterior and mediolateral data, the degree of fractal correlation is computed from a new time-series related to the absolute magnitude of COP displacement over time [16] [17].

It utilizes detrended fluctuation analysis (DFA) [18] to quantify fluctuation amplitudes after “detrending” (i.e., removing local trends) at different time scales. If a signal is fractal in nature, fluctuation amplitudes behave as a power-law function of time scale and the correlations of the signal can be characterized by a DFA-derived scaling exponent, α. Under healthy conditions, the value of α is typically close to 1.0 for many fractal physiological fluctuations, such as heart rate, motor activity and neural activity, indicating highly complex fluctuation patterns containing strong multi-scale correlations [19], [20], [21] [22] [23] [24]. Both aging and pathological conditions often disrupt these patterns, leading to alterations in the fractal scaling exponent [25].

In the original DFA method, local trends at different time sales are estimated by polynomial fitting [26] and the results are often affected by nonlinear filters [27], trends [28], [29] and nonstationarities [30]. In a recent study, Yeh et al [31] demonstrated that a modified DFA method in which local trends are determined by an adaptive data analysis technique termed empirical mode decomposition (EMD) [32] more reliably identifies trends in human heart beat dynamics as compared to traditional methods. The purpose of this study was thus to establish the relationship between COP complexity, as quantified by the EMD-DFA technique, and system adaptability in healthy younger and older adults. We tested the following hypotheses: 1) Aging from adulthood into senescence is associated with altered long-range correlations in COP magnitude fluctuations during quiet standing. We expected that α values would be greater in older as compared to younger adults. 2) The degree of long-range correlation within COP dynamics is related to the adaptive capacity of the postural control system. We therefore expected that α would be predictive of performance in the one-leg standing balance test, a widely-used clinical test of balance that identifies elderly persons at increased risk of future functional dependence and frailty [33].

## Materials and Methods

### Ethics

We think all the methods used in the experiment like force platform is very common without harm to body and the experiment process is also permitted in clinical like the Romberg test, which is very easy to take. This study and the consent procedure were approved by the ethics committee of Academy for Advanced Interdisciplinary Studies, Peking University.

### Subjects

Two groups of healthy adults subjects participated in the study: (1) 22 healthy young subjects aged between 21–25 years (11 men and 11 women, age = 23.45±1.34 years, height = 167.77±8.08 cm, body mass = 61.72±13.76 kg); (2) 22 older subjects aged 56–78 years (11 men and 11 women, age = 66.68±7.11 years, height = 168.68±7.36 cm, body mass = 66.31±10.05 kg). All subjects provided informed consent as approved by the local institutional review board. No subjects had cardiovascular, neurological, or other disorder that may influence movement. We obtained informed consent from all the subjects. All methods involved in this study achieved clinical acceptance and were presented to the subject in detail prior to obtaining consent.

### Experimental Procedure

Standing postural sway was measured for 20 seconds with a stationary force platform (Kistler Instrument Corp., Amherst, NY). Subjects stood barefoot with feet shoulder-width apart. Foot position was marked to ensure consistency between trials. Four trials were completed in random order: two with eyes open and two with eyes closed. Subjects were instructed to remain as still as possible throughout the trial.

Adaptive capacity of the postural control system was assessed with the sharpened Romberg test [34]. Subjects stood barefoot on their dominant leg for up to 20 seconds. The time to failure (i.e., when the dominant foot moved position or the non-dominant foot touched the ground) was recorded and averaged over two trials.

### Postural Sway Analysis

**Figure 1:**
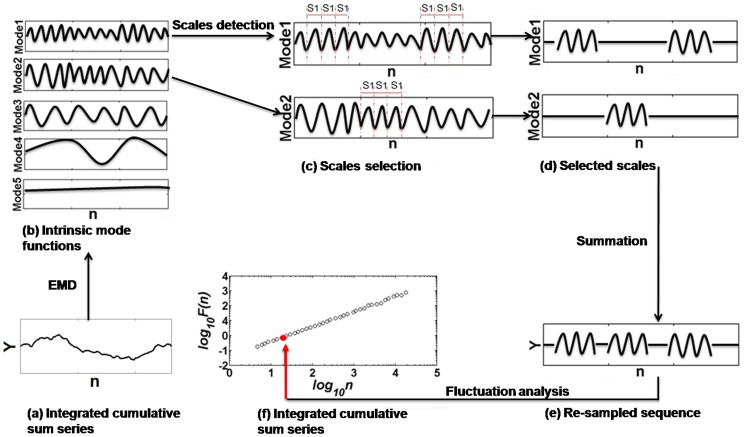
The empirical mode decomposition embedded detrended fluctuation analysis (EMD-DFA) technique.

Anterioposterior and mediolateral center-of-pressure displacements were recorded at 1000 Hz using Bioware software (Kistler Instrument Corp., Amherst, NY). A one-dimensional magnitude time-series was derived by calculating the absolute displacement between each sampled point. A one-dimensional time-series was derived by calculating the absolute displacement between each sampled point as following:
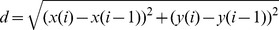
(1)where d is the magnitude (absolute displacement), and x (i) and y (i) are the coordinate value of current sampling point while x(i-1) and y(i-1) stand for the coordinate of the previous point. After that, instead of the original coordinate values in anteroposterior (AP) and mediolateral (ML) directions, the magnitude series could be gained, which was then analyzed by EMD-DFA method in a six step process (Figure 1).

A cumulative sum time series, *Y*(*i*), was constructed after removing the mean from the original time series *x*(*i*),*i* = 1,…,*N* (Figure 1a).

**Figure 2:**
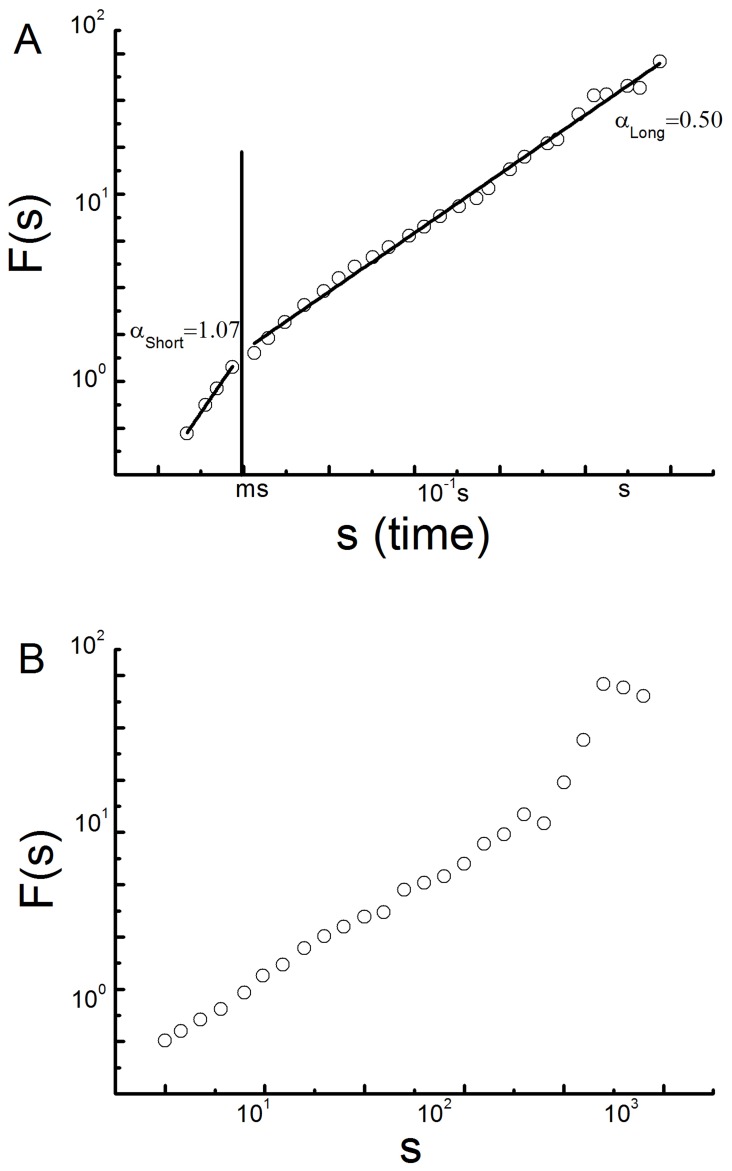
Comparison of A) EMD-DFA, and B) conventional DFA of standing postural sway dynamics (i.e., center-of-pressure) as a 23-year-old subject stood with eyes-open. Two distinct linear regions were present in the EMD-DFA-derived double log plot, thereby enabling calculation of a short- and long-term scaling exponent, α. The double log plot derived from the conventional DFA, on the other hand, did not contain a clear cross-over point. Similar results were observed for all analyzed datasets.

1. The data-driven EMD algorithm was applied to decompose the integrated time series *Y*(*i*) into a set of intrinsic mode functions, *IMF(1∼n)*, and a residual component (i.e., Mode 5, Figure 1b). The residual component, which represents a nonstationary trend, was removed.
2. All time scales inherent to the given COP series (i.e., intrinsic scale, *s)* were identified by computing the number of data points between each neighboring local minima throughout each *IMF (i.e.,
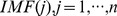
*). Each *s* was therefore mono-component, derived from the signal itself and not affected by nonstationary trends, thus reflecting an inherent property of the signal. Accordingly, all intrinsic scales of each IMF were determined (Figure 1c).
3. For each identified intrinsic scale (e.g.,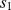), all IMFs were resampled such that zeros were substituted for all data points corresponding to scales not equal to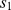. The fluctuation at the time scale *s*_1_ time-series, *Y_s_*_1_(*i*), was then generated by summing all *IMF_s1_*(*j*) (Figure 1e).
4. The root mean square, *F*(*s*_1_), of *Y_s_*_1_(*i*) was calculated using the following equation: 
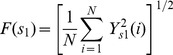
(2)
5. Steps 4 and 5 were repeated for all intrinsic scales, thus forming a power law relationship between 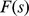 and *s*:
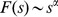
(3)where the scaling exponent α exhibits the scaling properties of the signal and can be estimated by the slope of a linear plot on a double log graph (Figure 1f).
6. For all datasets, two distinct linear regions were observed on the double log graph (Figure 2A). We therefore employed a least-squares fitting procedure to first determine the best cross-over point and then computed a short- and long-term alpha (α_st_. α_lt_).

For comparative purposes we also derived double log plots using the original DFA method. Similar to previous report [35], we did not observe a cross over point in any dataset and moreover, long-term scaling properties were highly unstable (Figure 2B).

In addition to the EMD-DFA method, traditional summary statistics were computed and averaged across trials. COP speed was computed by dividing total path length by trial duration. COP area was determined by calculating by area of a confidence ellipse enclosing 95% of the center-of-pressure trajectory [36].

### Statistical Analysis

Statistical analyses were performed using JMP software (SAS Institute, Cary, NC). Descriptive statistics (means ± S.D.) were used to summarize all numeric variables. Potential group differences in gender distribution, height and body mass were examined with one-way ANOVAs or logistic regression.

The effects of age on standing postural sway (i.e., α_Short_, α_Long_, speed, area) were examined with repeated-measures ANCOVAs. Group (i.e., young, old), visual condition (i.e., eyes-open, eyes-closed) and their interaction were included as model effects. Models were adjusted for gender, height and body mass. Tukey’s post-hoc testing was used to analyze group differences within significant models. As the four COP metrics were analyzed with a separate model, a Bonferroni adjustment of p<0.012 was used to determine significance.

The relationship between standing postural sway metrics and Romberg test performance (i.e., one leg standing time to failure) was examined using linear regression analysis. Models were adjusted for gender, height and body mass. As all the young subjects were able to complete the one-leg stand test without failing, relationships were only explored within the older group. Similar to above, significance was determined by p<0.012.

## Results

The younger and older groups did not differ in height, body mass or the distribution of gender.

With all subjects analyzed together, α_short_ and α_Long_ did not correlate with one another or with the traditional metrics of COP speed or area in either visual condition (r^2^<0.04, p>0.10).

**Figure 3:**
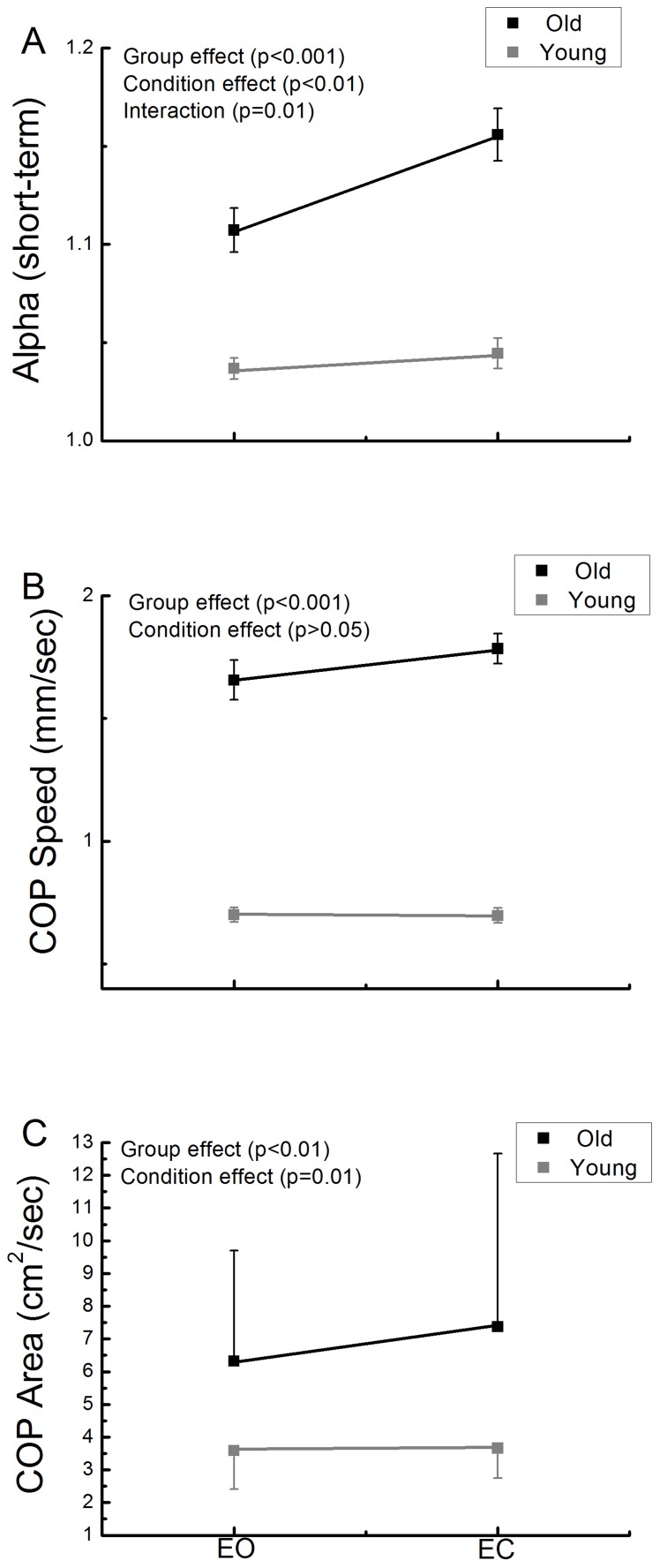
The effects of age and visual condition on postural sway metrics including A) the short-term scaling exponent (α_short_), B) center of pressure (COP) speed, and C) COP area. Values represent means ± standard error. The long-term scaling exponent (α_Long_) was similar between groups and across visual conditions and therefore not shown.

The long-term scaling exponent (α_Long_) was similar across groups and between visual conditions (0.46±0.12). On the other hand, an interaction (F = 5.3, p = 0.01) was observed between group and visual condition for α_Short_ (Figure 3a). As compared to the younger group, the older group demonstrated greater α_Short_ values when standing with eyes open and eyes closed. Occluding vision resulted in increased α_Short_ values across both groups; however, this effect was significantly greater in the older group (p<0.01).

The older group exhibited larger COP area (F = 4.5, p<0.01) and faster COP speed (F = 12.4, p<0.001) than the younger group (Figure 3 b and c). Closing the eyes led to an increase in COP area in both groups (F = 5.2, p = 0.01), yet did not affect COP speed.

**Figure 4:**
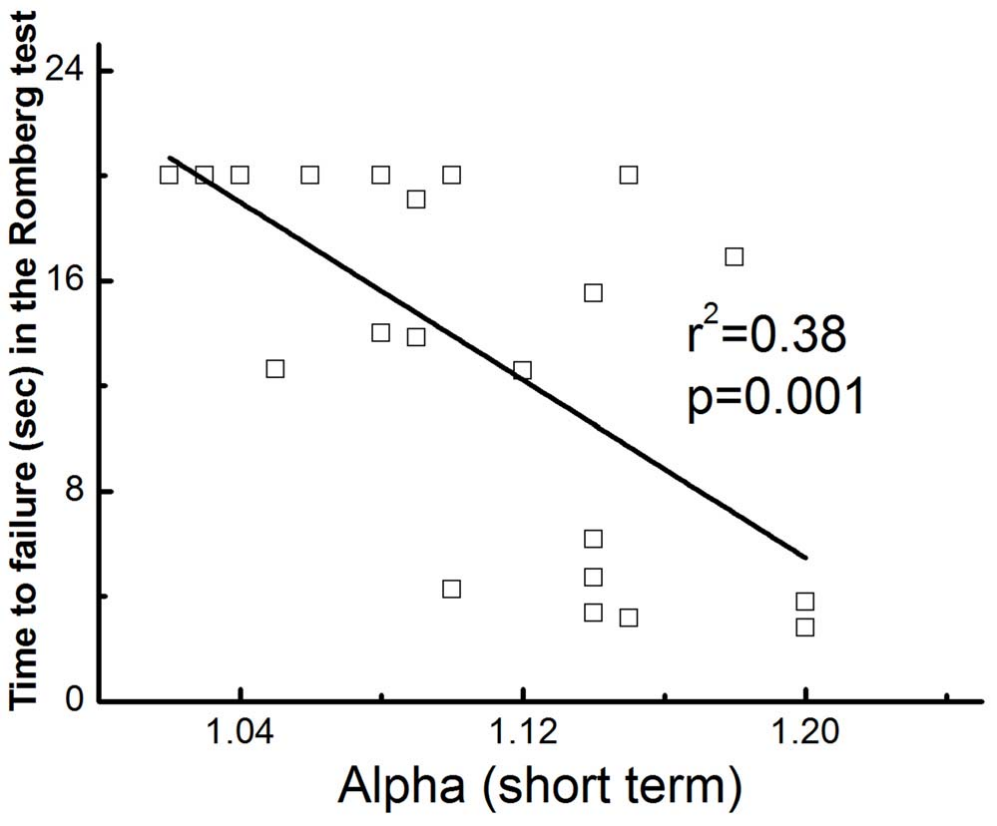
The relationship between the complexity of postural sway and the functionality of the postural control system in older adults. Those individuals with greater short-term COP complexity (i.e., EMD-DFA-derived α_Short_ values closer to one) during eyes-open standing demonstrated greater performance in the Romberg test of one-leg standing balance.

To examine the relationship between the degree of long-range correlations within COP dynamics and the adaptive capacity off the postural control system, we examined the relationship between COP metrics obtained during eyes-open bipedal standing and the time to failure in the single leg balance test. Only those data from older adults were utilized, as a ceiling effect occurred in the Romberg test in the younger group. Those older adults with lower α_Short_ values (i.e., closer to 1.0) were able to stand on one leg significantly longer (Figure 4, r = −0.64, p<0.001). This correlation remained significant (p = 0.01) after adjusting for gender, height and body mass. No other COP metric correlated with time to failure in the single leg test.

## Discussion

In this study, we applied EMD-DFA on to estimate the fractal-like complexity of standing postural control dynamics and its relationship to adaptive capacity in younger and older adults. Using this method, we have demonstrated that the fractal properties of COP magnitude fluctuations are time-scale dependent which is different from the raw data of COP. The α_Long_ parameter was nearly constant across all subjects in either visual condition which resembled white noise. On shorter time scales (less than 10 ms), however, fluctuations were highly complex. Biological aging and the removal of visual feedback were each associated with larger α_Short_ values; i.e., diminished short-term COP complexity. The value of α_Short_ was not correlated with traditional COP parameters and importantly, closely predicted system functionality in older adults as defined by performance in the Romberg test of one leg standing balance.

The EMD-DFA utilizes an adaptive decomposition algorithm to determine time scales that are intrinsic to the signal being analyzed. These intrinsic scales denote periods of mono-component oscillations and therefore, are not pre-determined or fixed periods, as is the case in the conventional DFA approach. This detrending procedure thereby affords more accurate identification (and removal) of intrinsic trends, as well as subsequent interpretation of the scaling properties associated with the complex control of standing postural sway. As opposed to conventional DFA, results derived from EMD-DFA consistently revealed two distinct linear regions on the double-log graphs, with an average cross-over point occurring at approximately 10 milliseconds. Similar to several previous studies on the analysis of the COPx and COPy data [19], [37], the short-term scaling properties of COP dynamics resembled pink noise (i.e., α_Short_ values were close to one). The long-term scaling properties were more stable than those derived from conventional DFA (i.e., Figure 2) and resembled white noise which is not similar to the result of original data in AP and ML.

In a previous study, Collins and De Luca [9] examined the COP dynamics of quiet standing using stabilogram diffusion analysis, a technique comparable to that of conventional DFA. Similar to the present study, the authors observed distinct short- and long-term fractal scaling characteristics. They concluded that in the short term (i.e., periods less than one second), COP fluctuations are dominated by persistent behavior and thus reflect open-loop control. Over periods greater than one second, on the other hand, COP fluctuations are anti-persistent, suggesting the presence of feedback-mediated control. In the present study, we observed a cross-over point that consistently occurred at approximately 0.01 second (for normal elder and young people it remains at the same time scale). While feedback-mediated reflexes do indeed occur on the millisecond level [38], the specific regulatory mechanisms that influence high-frequency COP dynamics are unclear. For example, in addition to studying feedback-mediated reflexes, future research should employ EMD-DFA to establish the influence of both muscle tone and joint stiffness characteristics on cross-over point timing, as each also influences the high-frequency components of standing postural sway [39].

Lipsitz and Goldberger proposed that aging from adulthood into senescence results in a loss of complexity associated with the dynamics of physiological control [40]. This concept has been supported by many studies. For instance, in a previous study of balance control, Thurner et al [41] calculated the power spectral exponent, β, of COP time-series acquired as healthy younger and older adults stood quietly with eyes open. The study showed that older adults exhibited higher β values over short time scales. As β is analytically related to α (β = 2α-1) [42], higher values of β reflect lower complexity of postural sway [43]. Our results show that the time courses of young are in higher anti-persistence than elderly (when 1<α<1.5, smaller α = more anti-persistent), which is correlated with a more tightly controlled postural system and higher balance stability [44], [45]. These results demonstrate a cross-sectional, aging-related degradation in the complex physiological control of standing posture.

Across all subjects, closing the eyes increased α_Short_ values and thus, reduced COP complexity. However, this reduction in complexity was significantly greater in older adults as compared to their younger counterparts. This observation supports the notion that with advancing age, the control of postural sway becomes increasingly dependent upon visual feedback [10], [46]. These results are also supported by Manor et al [25], who demonstrated–in a cohort of 453 community-residing elderly adults–that visual and somatosensory impairments were independently associated with diminished quiet standing COP complexity, as quantified by multiscale entropy analysis. Thus, as visual feedback appears to be involved in the complex regulation of postural control, particularly in elderly individuals, research examining the mechanisms through which this source of feedback contributes to the fractal-like nature of postural control across the lifespan is needed.

However, there are also studies showing that complexity in certain physiological variables is not necessarily reduced with healthy aging [47] [48] [49], and several caveats exist within this theory [50] e.g., the effects of biological aging may be dependent upon both the metric used to quantify complexity [51] and the task constraints within which a system is operating. Further studies are warranted to clarify potentially different influences of aging on different physiological systems and on different dynamic properties.

A central premise of the complexity theory of aging is that a loss of complexity in the dynamics of postural control results in functional decline of the individual by limiting the range of available, adaptive postural responses to the innumerable and often unpredictable stressors and perturbations experienced throughout one’s daily life [13], [40]. 1. Current study supports this premise that older adults with greater α_Short_ values of the magnitude series were able to stand longer on one-leg. 2. As Romberg test reflects function and fall risk, suggests that complexity is important and complimentary to traditional metrics. As we know the degeneration of balance control system resulting from the age increasing or disease leads to large amount of injuries and death in olds or patients, this finding may be helpful in falls predicting and protecting.

Of note, the fractal properties associated with the derived COP magnitude time-series may be different from those associated with the raw COP series. Previous studies have demonstrated that positive correlations in the magnitude series reflect nonlinear properties of the dynamics of physiological fluctuations, and that two signals can have similar correlations in raw data but different correlations in magnitude series and vice versa [16] [22] [52].

In this study, we examined COP complexity at time scales up to 20 seconds, which is relatively short. Further studies should be carried out to determine the EMD-DFA derived scaling properties of COP fluctuations at larger time scales. In addition, since this study was cross-sectional, future longitudinal studies are needed to examine intra-subject changes in postural sway complexity over time, as well as the relationship of these changes in complexity to clinical outcomes. Furthermore, to better understand aging effects on human movement in general, the EMD-DFA method can also be applied to other types of physiological signals such as wrist motion and gait, which also possess fractal and complex temporal fluctuations [2] [3] [52] [53] [54].

In conclusion, this study applied EMD-DFA to examine the aging and functional implications of the complex fractal properties of standing postural sway. The fractal-scaling exponents of magnitude series derived from this procedure were more stable than those produced by conventional DFA. The observed age-related reduction in COP complexity (i.e., larger α_Short_) was exaggerated by removing visual feedback, and was closely associated with diminished performance in the Romberg test; a widely-used clinical assessment associated with reduced mobility and elevated risk of falling. This property of postural control as a new biomarker may therefore aid in the evaluation of the postural control system and the identification of elderly people with functional limitations.
